# Home-cage behavior in the Stargazer mutant mouse

**DOI:** 10.1038/s41598-022-17015-3

**Published:** 2022-07-27

**Authors:** Catharina Schirmer, Mark A. Abboud, Samuel C. Lee, John S. Bass, Arindam G. Mazumder, Jessica L. Kamen, Vaishnav Krishnan

**Affiliations:** Department of Neurology, Baylor College of Medicine, One Baylor Plaza St, Neurosensory BCM: MS NB302, Houston, TX 77030 USA

**Keywords:** Circadian rhythms and sleep, Feeding behaviour, Learning and memory, Motivation

## Abstract

In many childhood-onset genetic epilepsies, seizures are accompanied by neurobehavioral impairments and motor disability. In the Stargazer mutant mouse, genetic disruptions of *Cacng2* result in absence-like spike-wave seizures, cerebellar gait ataxia and vestibular dysfunction, which limit traditional approaches to behavioral phenotyping. Here, we combine videotracking and instrumented home-cage monitoring to resolve the neurobehavioral facets of the murine Stargazer syndrome. We find that despite their gait ataxia, stargazer mutants display horizontal hyperactivity and variable rates of repetitive circling behavior. While feeding rhythms, circadian or ultradian oscillations in activity are unchanged, mutants exhibit fragmented bouts of behaviorally defined “sleep”, atypical licking dynamics and lowered sucrose preference. Mutants also display an attenuated response to visual and auditory home-cage perturbations, together with profound reductions in voluntary wheel-running. Our results reveal that the seizures and ataxia of Stargazer mutants occur in the context of a more pervasive behavioral syndrome with elements of encephalopathy, repetitive behavior and anhedonia. These findings expand our understanding of the function of *Cacng2*.

## Introduction

In patients with developmental and/or epileptic encephalopathies (DEEs/EEs)^1^, seizures and static intellectual disability are often associated with motor impairments (quadriparesis, ataxia), disturbances in sleep/arousal, sensory integration, feeding and emotional lability^2,3^. Behavioral comorbidities in particular amplify the risk of psychiatric side effects to antiseizure medications^4^, and compound polypharmacy through concurrent prescriptions for neuroleptics, psychostimulants and/or sedatives. With advances in mouse genome engineering, patient-informed genetic DEE mouse models now play a dominant role in devising precision genetic treatments, informed by the latest advances in transcriptomics, neuroimaging and neurophysiology^5–7^. In contrast, preclinical endpoints to ascertain neurobehavioral impairment have not experienced similar technical or conceptual innovations. Today’s most exciting mouse models often remain subject to a battery of maze- or arena-based assays applied *serially* (e.g., open field test, elevated plus maze, etc.)^8–10^. While enormously popular, these tests produce snapshots of behavior in singular readouts that may be amplified/attenuated by arena novelty, and which may be contaminated by variations in motor drive (e.g., hyperactivity) or function (e.g., ataxia)^11^. A growing movement seeks to adjunct these assays with naturalistic, continuous and unbiased assessments of spontaneous self-motivated behavior that can be analyzed across multiple time scales^12–15^. In this paradigm, prolonged experimenter-free home-cage recordings minimize observer effects^16,17^, and automated data collection enables computational phenotypes that can be appraised and compared without anthropomorphization^13,14,18^.

Here, we apply one such home-cage monitoring platform^12,19–21^ to visualize the extent and severity of behavioral impairment in Stargazer mutant mice^22^. These mutants arose from a naturally occurring disruptive transposon insertion in *Cacng2* encoding STARGAZIN, a transmembrane protein necessary for AMPA-subtype glutamate receptor expression. Characteristic 6–9 Hz spike/wave seizures in mutants beginning in adolescence^22–25^ have been linked to reduced AMPAR expression in inhibitory neurons of the reticular thalamus^26,27^. Mutants also display gait ataxia, sustained extensor movements of the neck, severely impaired rotarod performance and are unable to swim^28^: these motor and vestibular phenotypes have been linked to diminished AMPAR expression in cerebellar Purkinje cells^27^ and/or vacuolar degeneration of the vestibular epithelium^28^. Using prolonged experimenter-free recordings and provocative maneuvers applied *within* the home-cage, we sought to examine whether Stargazer mutants display features of neurobehavioral impairment that are distinct from (and comorbid with) their known motor and epilepsy phenotype.

## Results

**Figure 1:**
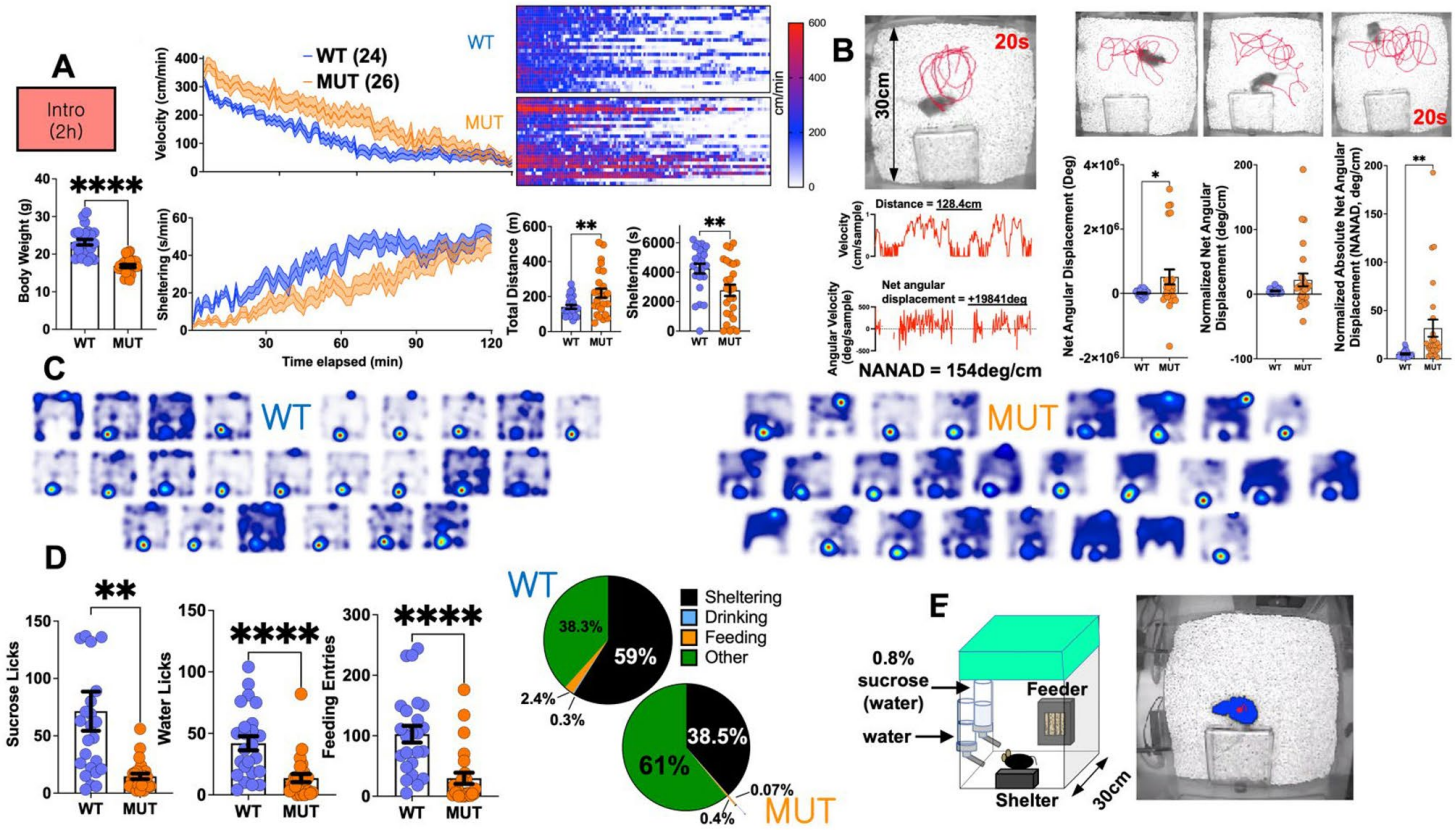
Habituation to the home-cage. (**A**) In a 2 h-long introductory trial, mutants displayed hyperactivity and reduced shelter engagement. TOP RIGHT: Raster plot of individual velocities in cm/min for all mice. (**B**) Some mutants displayed repetitive circling behavior. LEFT: An illustration from a circling mouse depicting a 20 s-long track, where a horizontal displacement of ~ 128 cm is associated with a ~  + 19,841 deg angular displacement (signed + ve for counterclockwise turns), resulting in a [distance] Normalized Absolute Net Angular Displacement (NANAD) of ~ 154 deg/cm. RIGHT: Averaged across the entire trial, mutants displayed significantly higher NANAD values. (**C**) Heat maps of position probability during the 2 h trial confirming robust cage exploration in both groups. (**D**) Mutants accumulated significantly fewer licks and feeding entries, alternatively visualized as time budgets (right). (**E**) Home-cage schematic and representative aerial view with centerpoint (red) and body contour (blue). Mean ± s.e.m shown.

On admission to home-cages at ~ p50, mutants were comparatively underweight. Their initial habituation response was marked with hyperactivity and reduced shelter engagement (Fig. 1A). A wobbling titubating gait was plainly evident. Some mutants engaged in repetitive circling behavior (Supplementary Movie 1), producing circular tracks of varied diameter that were not necessarily concentric. This resulted in an overall increase in *positive* total net angular displacement (NAD), reflecting a preference for counterclockwise circling. Absolute NADs remained significantly higher in mutants even after normalizing by total distances (NANAD, normalized absolute net angular displacement), with mutants accumulating a net rotation of ~  ± *31 deg* for every centimeter of horizontal displacement (vs *4.9 deg/cm* in WT mice, Fig. 1B). While both groups thoroughly explored non-shelter regions (Fig. 1C), mutants also displayed a relative avoidance of waterspouts and their food hoppers (Fig. 1D,E). Together, these results define the initial mutant response to an enriched novel cage, marked by hyperactivity, repetitive circling behavior and novel object avoidance.

**Figure 2:**
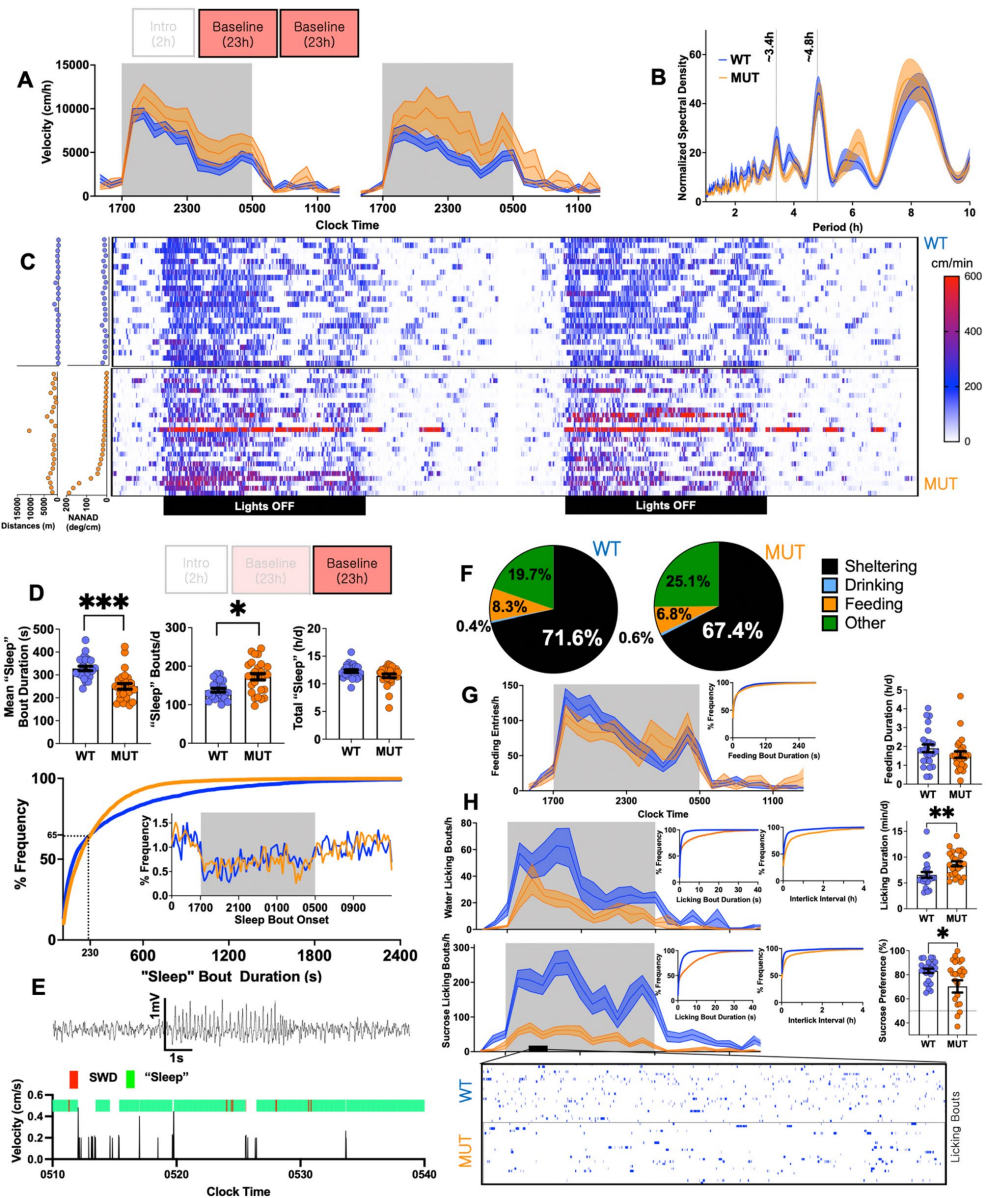
A representative day. (**A**) During 2 consecutive 1400–1300 baseline recordings, mutants navigated greater horizontal distances. (**B**) Lomb-scargle periodogram of ultradian oscillations in locomotor activity depicting peaks at harmonic frequencies of the circadian oscillator. (**C**) Raster plot of individual velocities over two baseline recordings. Hyperactivity was not directly related to high measures of circling (NANAD). (**D**) Mutants displayed fewer but longer “sleep” bouts without affecting total daily sleep. BOTTOM: Frequency distribution histogram comparing “sleep” bout durations and bout onset (inset). (**E**) Wireless EEG conducted within home-cages revealing an example of how spike/wave discharges occurred within behaviorally defined “sleep” bouts. (**F**) Daily time budgets, revealing lower shelter times and increased “other” behavior. (**G**) Mutants exhibit total feeding times, feeding entries and bout durations that are similar to WTs. (**H**) Mutants accomplish fewer but longer licking bouts of sucrose or water. Separately, mutants also displayed an overall reduction in sucrose preference. Mean ± s.e.m shown.

During subsequent baseline recordings, mutants remained hyperactive predominantly during the nocturnal phase, with a similar spectral distribution of ultradian oscillations in locomotor behavior (Fig. 2A,B). Raster plots of velocity binned minutely revealed generally similar asynchronous and “bursty”^15^ patterns of rest and activity in both groups, with greater synchrony at dark–light transitions. Measures of circling behavior poorly correlated with total daily distances (Fig. 2C). To more granularly examine changes in arousal, we applied an established and validated noninvasive method which estimates “sleep” (enquoted to emphasize our behavioral definition) as periods of sustained immobility lasting ≥ 40 s^19,20,29–32^. Under these criteria, mutants displayed shorter and more frequent bouts of “sleep”, without an overall change in total “sleep”. These differences primarily affected “sleep” bouts longer than ~ 230 s, while the timing of “sleep” bouts throughout the day was unchanged (Fig. 2D). In a separate group of mutant mice monitored wirelessly with EEG, we observed that spike/wave seizures were typically brief (2–6 s) and were frequently encountered during (without prolonging) bouts of “sleep” (Fig. 2E).

Next, we tallied total durations of licking, feeding and sheltering during the second baseline recording to visualize averaged daily time budgets^33^. Mutants displayed a trend towards lower sheltering times (p = 0.1), which could not be explained by increased feeding (Fig. 2F,G). Lickometry data revealed major abnormalities in the structure of fluid intake, with mutants accomplishing *greater* overall licking durations through a combination of *fewer* but *longer* licking bouts. Simultaneously, while WT and the vast majority of MUT mice preferred sucrose water over water, mutants displayed a significantly lowered sucrose preference (Fig. 2H). Across these various phenotypes, no significant gender-based distinctions were identified (Supplementary Fig. 1). Collectively, these baseline data highlight a set of diffuse alterations in the spatiotemporal structure of spontaneous behavior in mutant mice.

**Figure 3:**
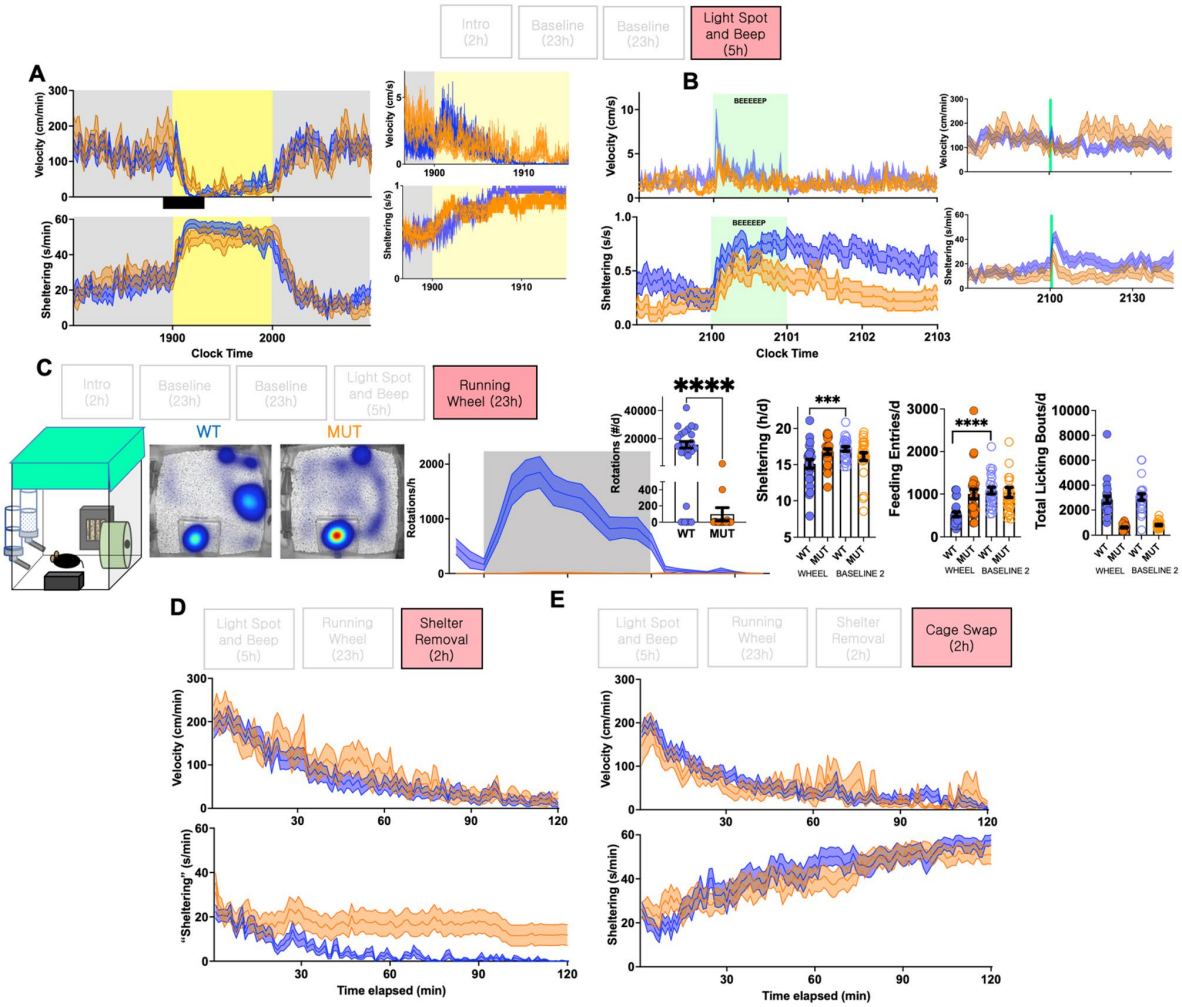
Provocative maneuvers. (**A**) Responses of WT and mutants to the “light spot test”. (**B**) Mutants display an attenuated early locomotor startle and sheltering response to a 60 s long home-cage beep stimulus. (**C**) Mutants display a significant reduction in wheel-running behavior. During this trial, WT mice display a relative suppression of sheltering and feeding behavior (compared with data from baseline day 2, two-tailed *paired* Student’s t tests). (**D**) Locomotor and sheltering response to shelter removal, and (**E**) cage swap protocol. Mean ± s.e.m shown.

We began our provocative maneuvers with an hour-long “light spot test”^19,20,34^, which poses a conflict between nocturnal foraging and light aversion within their home-cage and without human presence. WT mice responded acutely with sharply *increased* activity followed by sustained immobility within shelters. In comparison, a relatively blunted locomotor suppression response was seen in mutants, with some minimal persistent exploration of the open illuminated cage (Fig. 3A). Similarly, in response to a 60 s-long pure tone, mutants exhibited an attenuated startle and sheltering response compared with WT mice (Fig. 3B). On the following day, when provided access with a running wheel, wheel-running behavior was almost entirely absent in mutants, with only 3/26 mutants successfully engaging the wheel. Compared with baseline recordings, wheel access suppressed feeding and sheltering in WT mice, an effect that was absent in mutants (Fig. 3C).

Finally, we applied two additional brief daytime perturbations. Since mice spend the bulk of their light period/daytime within shelters^19,20^, we examined their response to transient shelter removal. While WT mice settled in cage corners, many mutants rested in the cage location previously occupied by the shelter (Fig. 3D). In a second daytime maneuver, mice were manually repositioned into a cage previously inhabited by a gender-matched mouse ("cage swap"), providing a geometrically similar field with novel olfactory cues. Unlike their initial habituation response (Fig. 1A), activity patterns and gradual shelter entry were similar (Fig. 3E).

## Discussion

Advances in wearable technologies are poised to transform the diagnosis and management of mental health disorders by supplementing subjective and qualitative clinic-based assessments of mental status with more continuous, in situ and quantitative device-based measurements^35^. Home-cage measures of mouse behavior provide a preclinical platform to align basic neuroscience with the digitization of neuropsychiatric symptoms. By incorporating a wide array of endpoints, this approach can identify murine behavioral symptom constellations that may not (and need not) perfectly parallel human DSM-defined syndromes (e.g., autism spectrum disorder), and which may be unique to specific etiologies of pervasive neurodevelopment. For example, we have shown using our home-cage chambers that mice with *Scn1a* mutations^36^ (modeling Dravet syndrome) display nocturnal hypoactivity, hypersomnia and hypodipsia^20^, while mice prenatally exposed to valproic acid exhibit hypophagia and increased wheel-running without changes in sleep and activity.

Here, we adopted a similar approach to examine home-cage behavioral abnormalities in Stargazer mutant mice, recognizing that any automatically gathered videotracking or “metered” data (e.g., lickometers, feeding meter) must be appraised in the context of this mutant’s known neurological impairments. While we expected that a combination of gait ataxia and spike/wave seizures would at least somewhat impede movement, mutants displayed *increased* activity levels when first introduced into home-cages, as well as nocturnal hyperactivity during prolonged unperturbed baseline recordings. Simultaneously, both the suppression of movement by unexpected bright light, and a startle-like response to an unexpected tone stimulus were blunted in mutants. These findings suggest either diminished vigilance or indifference, which may directly relate to the burden of ongoing and/or cumulative epileptiform discharges. Interestingly, locomotor responses to daytime perturbations that necessitated brief human exposure (shelter removal, swap) were similar between groups.

Mutants also displayed shorter and more frequent bouts of “sleep”, which we estimated noninvasively by tallying epochs of sustained immobility ≥ 40 s in duration^29^. We acknowledge that this algorithm fails to distinguish between REM (rapid eye movement) sleep, non-REM sleep or quiet wakefulness. While spike-wave seizures that interrupt active wakefulness can cause paroxysmal arrests of movement, these events are typically much shorter in duration (2–6 s on average^22,37,38^) and would not have been registered by our algorithm’s cutoff. Average NREM or REM sleep bouts in mice are significantly more prolonged (180–360s or 60–120s^39–42^, respectively). Further, in patients with genetic or symptomatic generalized epilepsies, spike-wave discharges commonly occur during NREM sleep^43^, where they often persist in patients who are clinically seizure-free. While this could theoretically result in more protracted “sleep” bouts, we observed the opposite phenotype. Prolonged and detailed gold-standard multichannel EEG-EMG recordings, ideally deployed wirelessly within similar chambers, are clearly necessary to definitively confirm these changes in sleep and arousal patterns, as well as clarify the state dependence of spike-wave discharges in this model.

About a third of mutant mice displayed repetitive circling behavior^28^, which we depicted as a continuous variable by tallying angular displacement (NANAD). Circling is commonly observed in rodents with asymmetries in nigrostriatal dopamine signaling, vestibular disease^44^, and in several genetic mouse models of autism spectrum disorder^45^, where it may represent an analog of repetitive behaviors. One previous report identified features of vacuolar degeneration within the vestibular epithelia of the semicircular canals in Stargazer mutants, which correlated with abnormal behavioral responses to experimentally applied vestibular stressors, such as rotarod testing, intermittent horizontal spinning or upside down inversion^28^. One possibility is that the variable penetrance of the circling phenotype measured in our study (in the absence of any imposed vestibular stress) may relate to inter-individual variations in the severity or asymmetry of semicircular canal pathology.

Through lickometry, we also observed that mutants displayed polydipsia, with *more* prolonged but *less* frequent licking bouts. No such differences were identified in the temporal structure of feeding bouts. Abnormal licking patterns may reflect primary deficits in cerebellar coordination (*licking ataxia*) and/or an underlying limbic disturbance in consumptive behavior (*psychogenic polydipsia*^46^), which may also be driven by cerebellar influences on forebrain function^47,48^. While abnormal licking patterns were seen at both spouts, Stargazer mutants displayed a significantly lowered overall preference for sucrose water. This result points to a concurrent anhedonia, with the caveat that *Cacng2*’s role in primary taste perception remains unknown. Finally, the vast majority of mutants (23/26) did not engage with a running wheel. This may relate to anhedonia *and* a combination of vestibulopathy and gait ataxia (although 3/26 mutants were able to successfully achieve an average of ~ 830 rotations).

It remains to be determined whether some or all of these phenotypes may remit with early long term antiseizure medications, which can themselves impact home-cage behavior in wild type mice (e.g., valproic acid, which robustly increases wheel running^19^). In one previous study, ethosuximide treatment successfully suppressed spike/wave seizures in Stargazer mutants without impacting underlying aberrant patterns of EEG phase-amplitude coupling^23^, providing at least one correlate of a static network derangement that is unaffected by temporary seizure suppression. Ultimately, distinguishing those neurobehavioral consequences that are directly related to *Cacng2* loss from those which are secondary to epilepsy in Stargazer mutants remains both a theoretical and practical challenge. Conditionally ablating *Cacng2* in adult mice (e.g., via tamoxifen-mediated recombination^49^) could potentially create a narrow window for behavioral evaluation during which genetic loss of function may not be accompanied by spike/wave discharges. However, tamoxifen exposure itself may cloud such results. *CACNG2* genetic variants have been identified in small cohorts of patients with schizophrenia and bipolar disorder (without epilepsy or ataxia)^50–53^, but such variants have not met genome wide significance^54,55^.

In conclusion, our study provides a behavioral characterization of the Stargazer mutant mouse using a home-cage approach that can be applied broadly to study mice that model a triad of seizures, neurobehavioral impairment and motor abnormalities. With similar analyses, future studies may discern how these behavioral endophenotypes may be affected in mutants with graded variations in *Cacng2* expression (*Stargazer-*3 J mice, *Waggler*) or in mutants with etiologically distinct syndromes of ataxia and absence seizures (*lethargic [Cacnb4], ducky [Cacna2d2], tottering [Cacna1a]*)^56^.

## Methods

Protocols were approved by the Baylor College of Medicine Institutional Animal Care and Use Committee and conducted in accordance with USPHS Policy on Humane Care and Use of Laboratory Animals. We confirm that study details are provided in accordance with ARRIVE guidelines. Heterozygous mutant mice on a C57BL/6 J background^23,24,57,58^ were bred to obtain wildtype (WT) and homozygous mutant mice. PCR-based genotyping was performed on tail DNA at ~ p16, and mice were weaned into gender-matched cages at p21. No mice were excluded. Between ~ p21–28, all weaned cages were provided with Bio-Serv Nutragel as to supplement nutrition to mutants in the setting of ataxia and low body weight. At ~ p50, mice were transferred to Noldus Phenotyper home-cages (30 × 30 × 47 cm) within a designated satellite study area^19,20^. A total of 16 home-cages were employed in groups of four (“quad units”), located on two shelves of two wireframe racks. Each cage contains (i) two lickometered water sources (0.8% sucrose-drinking water Vs drinking water), (ii) an infrared (IR)-lucent shelter, an aerial IR camera and IR bulb arrays, (iii) a beam-break device to measure entries into a food hopper, (iv) a detachable running wheel, and (v) a 2300 Hz pure tone generator and an LED house light. Satellite temperature (20–26C), humidity (40–70%) and light cycle settings (ON between 0500–1700) matched vivarium conditions. White noise was played continuously, and satellite access was restricted to gowned, gloved, masked and capped personnel to minimize olfactory variations. Mice were distributed randomly to home-cages ensuring that one gender or one genotype was not over-represented within a single quad unit. When conducting within-cage daytime tasks described (positioning running wheel, removing shelter, etc.), operators were blinded to genotype.

Live videotracking (Noldus Ethovision XT14) sampled object x–y location (“centerpoint”) at 15 Hz, providing time series data for arena position (heat maps, shelter time), horizontal displacement (velocity) and relative angular velocity (*positively* signed for counterclockwise turn angles). “Sleep” epochs were defined as contiguous periods of immobility lasting ≥ 40 s, previously validated to provide > 90% agreement with neurophysiologically-determined sleep^29–32^. WT (n = 24, 9 female) and littermate mutants (n = 26, 16 female) obtained from multiple litters were studied in cohorts of 8–12 mice. Core phenotypes, separated by sex, are available in Figure S1. A modular design^19^ was applied beginning with a 2 h-long initial habituation study (“Intro”) and two consecutive 23 h long baseline recordings each beginning at 1400. Then, we applied visual and auditory stimulation (“light spot^19,20,34^ and beep^19^”), followed by a third prolonged recording in the presence of a running wheel. We concluded with two daytime provocations to interrogate their response to transient removal of their shelter (“shelter removal”) and a novel cage stress (“cage swap”).

For simultaneous electroencephalography (EEG), six ~ 7-week old mutants were implanted with EMKA easyTEL S-ETA devices under sterile precautions and isoflurane anesthesia. Biopotential leads (2) were affixed subdurally in right frontal and left posterior parietal regions using dental cement, with wires tunneled to a transponder positioned in the subject’s left flank. Wireless EEG was acquired at 1000 Hz sampling rate with IOX2 software (EMKA Technologies) via easyTEL receiver plates placed underneath home-cages, and EEG signals were inspected with LabChart reader using a bandpass filter (1–30 Hz). Seizures were defined as bursts of 7–9 Hz spike/wave discharges with an amplitude at least 2 × baseline voltage^57^. Data were graphed and analyzed with Prism Graphpad 9, always depicting mean ± standard error of the mean. Lomb-scargle periodograms (Matlab) were applied to calculate the power and peaks of ultradian oscillations in activity. Unless otherwise specified, two-tailed, unpaired student’s T tests were applied, with *, **, ***, **** depicting p < 0.05, < 0.01, < 0.001 or < 0.0001 respectively.

## Supplementary Information

Supplementary Information 1.

Supplementary Video 1.

## Data Availability

Raw data, video and EEG recordings from the current study are available from the corresponding author upon request.
