# Supplementary material for: Home-cage behavior in the Stargazer mutant mouse
Source: Sci Rep. 2022 Jul 27;12:12801. doi: 10.1038/s41598-022-17015-3 (PMC9329369; doi:10.1038/s41598-022-17015-3)
Supplement: Supplementary file 1 — Supplementary Information 1. [file 41598_2022_17015_MOESM1_ESM.docx]

**Supplementary File**

**Homecage Behavior in the Stargazer Mutant Mouse**

Catharina Schirmer, Mark A Abboud, Samuel C Lee, John S Bass,

Arindam Ghosh Mazumder, Jessica L Kamen, Vaishnav Krishnan

Department of Neurology

Baylor College of Medicine

Houston, TX USA

Corresponding Author:

Vaishnav Krishnan MDPhD

One Baylor Plaza St

Neurosensory BCM: MS NB302

Houston, TX: 77030

United States of America

[vkrish@bcm.edu](mailto:vkrish@bcm.edu)

Ph: 17137982238

Fax: +17137987561

**Supplementary Movie 1**: Representative recordings from WT (top and bottom left) and MUT (top and bottom right) mice captured during the introduction trial. The mutant mouse on the bottom right engages in repetitive circling behavior ~35s into the recording.

**
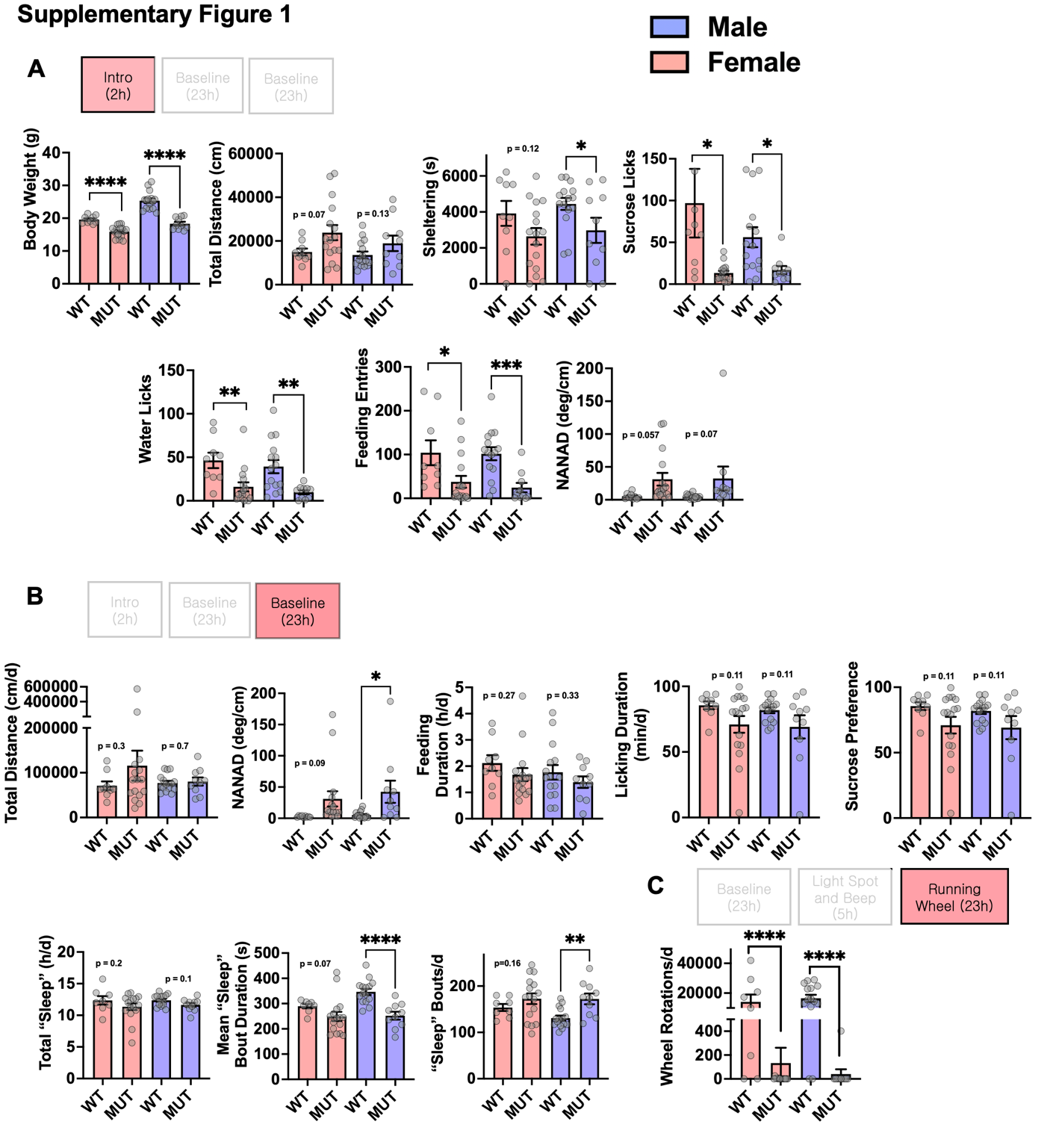
**

**Supplementary Figure 1.** Gender Differences in Neurobehavioral Phenotypes observed during the introductory trial (A), the second baseline day (B) and wheel-running trial (C) . Mean + s.e.m shown.
